# ENUCLEATION IN MALIGNANT CHOROIDAL MELANOMA - results in 15 years of using a new material in the prosthesis of the orbital cavity

**Published:** 2012-06-18

**Authors:** CP Tataru, MD Pop

**Affiliations:** Ophthalmology Department, “Carol Davila” University of Medicine and Pharmacy, Bucharest

**Keywords:** Choroidal malignant melanoma, enucleation, Polyethylene terephthalate, Dacron

## Abstract

Rationale: Enucleation implants are covered with a material that allows the fixation of the extraocular rectus muscles. Usually, the implants are covered in donor sclera, which implies the risk of infection transmission, inflammation and implant rejection, being also an expensive procedure. The new materials used for implant meshing should be tested and a safer and cost effective solution should be researched.

Objective: This study presents the results obtained after a 15-year use of an original prosthesis for the reconstruction of the orbital cavity after enucleation surgery.

Methods and results: 42 eyes of 42 patients who underwent enucleation surgery for choroidal malignant melanoma were included in the study. The surgical technique was very similar to the classic enucleation, the major difference being the implant of a prosthesis made out of a Polymethyl methacrylate (PMMA) ball covered by a Polyethylene terephthalate (dacron) shell used in cardiovascular surgery. All the patients had a very good technical result, without the inflammation of the orbital cavity, conjunctiva or eyelids, which demonstrates a very high material tolerability and an excellent cosmetic result. Late implant expulsion appeared in 7.14% of the patients (3 cases).

Discussion: The particularly good results obtained by using this technique, the absence of an inflammatory reaction after surgery, and the long lasting stability of the implant, recommend the method as being safe, with no major complications and a good esthetic result.

**Abbreviations**Polymethyl methacrylate (PMMA), Malignant choroidal melanoma (CMM)

## Introduction

Uveal melanoma is a malignant intraocular tumor, originating in the ectoderm melanocytes. The incidence of the tumor is of 1:150000-1:200000 in white people, mostly in men, being the most common primary intraocular tumor in adults. It primarily affects patients in their 50s and 60s, being extremely rare in children [**15**]. The tumor has a predilection for lightly pigmented individuals, blue eyes, and blonde hair. The etiology is unknown. No genetic associations or risk factors have been conclusively linked to the disease, apart from chronic sun exposure [**19**].

Choroidal malignant melanoma (CMM) has a high capacity of hematological dissemination to the liver, lungs, bones, kidneys, brain. The tumor size represents the most important indicator of vital prognosis and survival rate. Even if enucleation is performed in due course, half of the patients who undergo surgical treatment for CMM die from complications of subsequent metastasis [**4**].

**Fig. 1:**
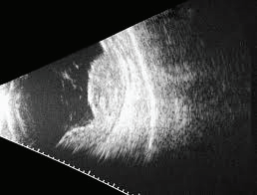
Choroidal malignant melanoma - B scan ultrasonography

**Fig. 2:**
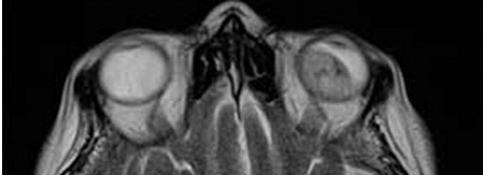
Choroidal malignant melanoma - magnetic resonance imaging

According to their location, uveal melanomas can be anterior (iris melanoma) or posterior (choroid and ciliary body melanoma **Fig. 1**, **Fig. 2**). The tumor can affect more than one uveal structure at the same time [**19**].

Most patients with CMM are asymptomatic, the tumor being discovered on a routine ophthalmological examination [**19**].

Small iris melanoma can be observed periodically. Larger or fast growing lesions should be excised (iridectomy or iridociclectomy) [**7**]. Enucleation is reserved for large or diffuse lesions, extension through sclera, and painful eye with no perception of light secondary to complications [**11**].

Melanoma of the ciliary body and choroid should always be treated. Until 1960, enucleation was the usual treatment. Nowadays, due to the development of more conservatory techniques (radiotherapy, application of radioactive plaque to the sclera, charged-particle radiation, excision with Gamma knife, endoresection, external transscleral resection, laser therapy, cryotherapy, chemotherapy) its incidence dropped significantly, but still remains the therapeutic solution for eyes with large tumors or blind and painful from tumor complications [**6,11**].

The Collaborative Ocular Melanoma Study (COMS) [**18**] (which compared enucleation with enucleation preceded by external-beam radiotherapy in patients with CMM) set up the following therapeutic recommendations:

- radiotherapy for small tumors (longitudinal diameter 1-3 mm, anterior-posterior diameter >5 mm)

- enucleation for medium (longitudinal diameter 2.5-10 mm, anterior-posterior diameter <16 mm) and large tumors (longitudinal diameter >10 mm; anterior-posterior diameter >16 mm).

In the absence of detectable metastasis, enucleation is done for curative purpose [**1**]. Nevertheless, after the procedure, the surgeon is left with the challenge of improving the cosmetic appearance of the patient. A perfect result implies a filled orbital cavity and a naturally looking prosthesis that moves symmetrically with the fellow eye.

The design of orbital enucleation implants has evolved significantly and there are two types of implants: non-integrated (non-porous) and integrated (porous) implants. Non–integrated implants (such as PMMA) do not allow the in-growth of organic tissue into their inorganic substance [**13**]. The porous nature of integrated implants (Hydroxyapatite, aluminium oxide, and polyethylene) allows ﬁbrovascular in-growth throughout the implant [**5**]. Enucleation implants are covered with a material that allows the fixation of the extraocular rectum muscles, thus permitting eye movement. The majority (59%) of the ophthalmic plastic surgeons use donor sclera when placing an intraorbital implant [**8**], which implies the risk of infection transmission, inflammation, implant rejection [**12**], being also a high costing procedure. New materials used for implant meshing are in research and a safer and cost effective solution should be developed.

The originality of our technique is the use of a new material - Polyethylene terephthalate (Dacron) - as a shell for the PMMA ball [**2**]. This is a thermoplastic polymer resin of the polyester family. Dacron is a synthetic material used with good results in cardiac and vascular surgery for making grafts and suture threads. We used it both as tubular material (like arteries which, after the ball insertion, were sutured at both heads) and as dacron tissue which entirely covered the PMMA ball. All the sutures with dacron were done with non-resorbable thread.

Dacron has the following characteristics: pores with a maximum diameter of 10 microns, Rockwel toughness of M94.-101, tension module of 2- 4 GPa, tension power of 80 Mpa, density of 1.3-1.4 g/cm3 , water absorption after 24 h of 0.1% and thermal conductivity of 0.15 – 0.4 W/mK [**9**].

## Methods

The study includes 42 eyes (42 patients, 23 females and 19 males) that underwent enucleation for malignant choroidal melanoma, histologically confirmed, between June 1996 and March 2012. Two surgeons using the same technique performed the surgeries.

**Fig. 3a and Fig. 3b:**
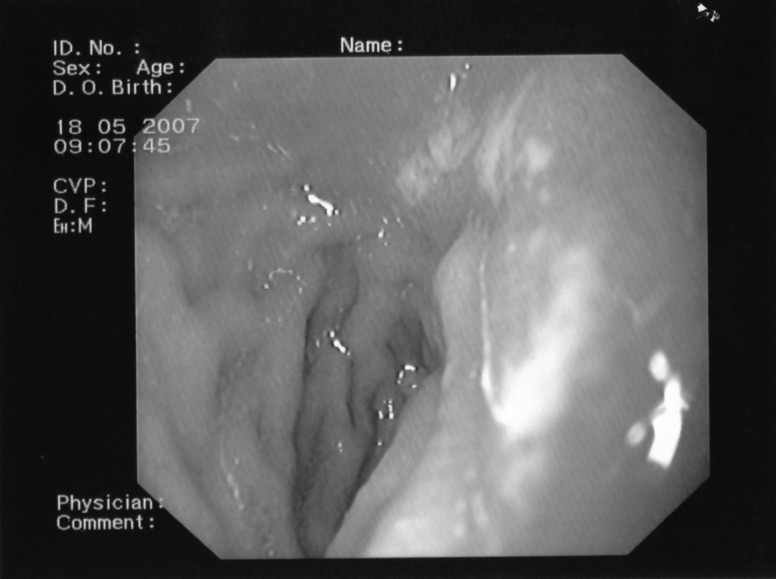
Catch of the muscles with 5.0 non-resorbable suture thread and their resection near the insertion; Eye prosthesis made up of a Polymethyl methacrylate) (PMMA) ball covered with a Polyethylene terephthalate shell (Dacron)

**Fig. 4:**
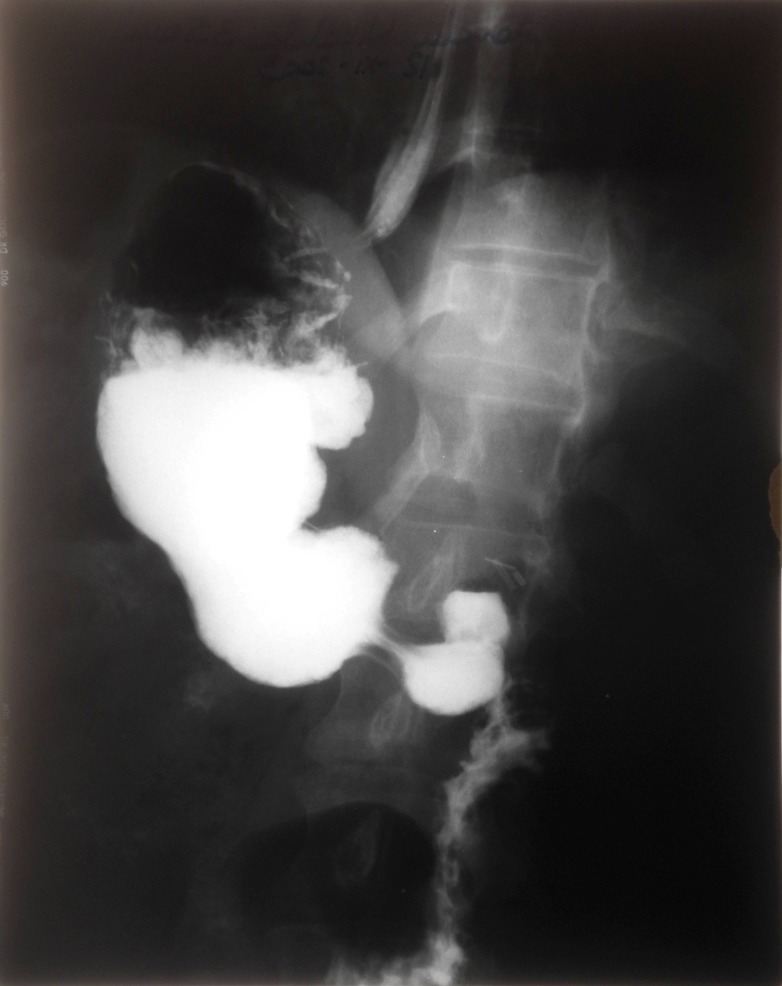
Reinsertion of the eye’s rectus muscles to the dacron shell with non-resorbable threads

The surgical times of this technique are:

- Decollation of the conjunctiva at the sclerocorneal limbus, on approximately 10 mm.

- Highlight of the eye’s rectus muscles with the help of strabismus’ hooks, catch of the muscles with 5.0 non-resorbable suture thread and their resection near the insertion (**Fig. 3a**) [**10**].

- Cut of the superior and inferior oblique muscles.

- At the insertion of the inferior oblique, a plier is attached; it helps lift the globe in order to cut the optic nerve.

- Cut of the optic nerve within the safety oncological limits (at 1-2 mm from the sclera) using a heavier enucleation scissors and removal of the globe with an enucleation spoon.

- Insertion of an eye prosthesis made up of a Polymethyl methacrylate) (PMMA) ball covered with a Polyethylene terephthalate shell (Dacron) (**Fig. 3b**) [**10**].

- Reinsertion of the eye’s rectus muscles to the dacron shell with non-resorbable threads (**Fig. 4**) [**10**].

Take off the tenon from the conjunctiva and suture in two plans, one for tenon, and one for the conjunctiva, with a 6.0 absorbable thread.

**Fig. 5:**
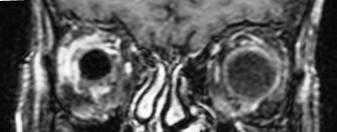
RE-MRI aspect after PMMA ball-dacron surgery

**Fig. 6:**
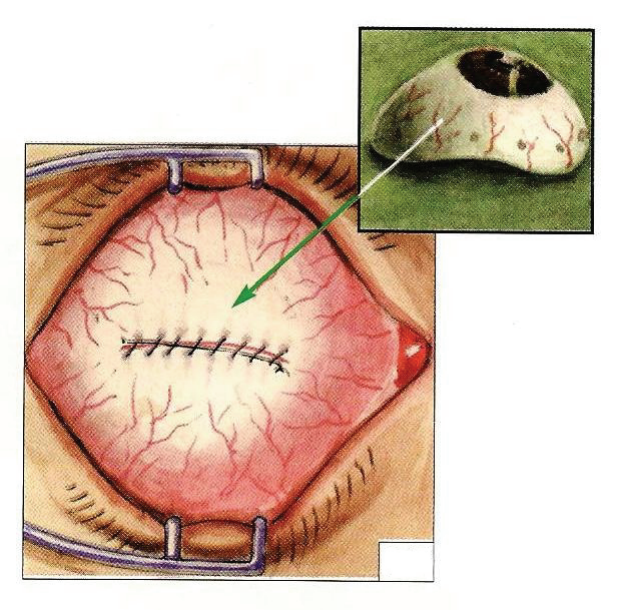
Final prosthesis

After surgery, the patients were followed up at 1, 4, 12 weeks (**Fig. 5**), 6 months and annually, in order to discover the possible complications. The final prosthesis was applied 6-8 weeks after the surgery (**Fig. 6**).[**16**]

## Results 

The maximum length for the follow up of the patients was of 15 years (2 cases over 13 years). In 3 cases (7.14%), the late expulsion of the ball appeared and surgical reintervention was needed in order to restore the orbital cavity. In comparison with the previous data about complications after enucleation and the insertion of a prosthesis ball made of hydroxyapatite, a lower frequency was discovered (Suter et all. reports 19.3% of the minor ball exposure and 7.5% expulsion, which needed reintervention from the total number of 120 patients during 8-9 years) [**14**].

All the patients had a very good technical result, without any early swallowing of the orbital cavity, conjunctiva or eyelids, which demonstrates a very good material tolerability and an excellent cosmetic result.

The dacron mesh for the orbital implant had a well tolerated motility without a high rate of extrusion and infection. Dacron use offered a long lasting material and reduced the costs compared to a sclera-covered implant.

## Conclusions

This technique is easily accessible to ophthalmology surgeons, the insertion is relatively facile and the costs are low [**17**]. A significant advantage over the hydroxyapatite implant is that they are far more affordable [**8**]. An important gain of the technique is a good prosthesis motility offered by the insertion of rectus muscles to the dacron shell. Nevertheless, the motility obtained after inserting a hydroxyapatite ball is superior to the dacron technique because of the transmission of the “epy-prosthesis” movement through the articulation mode [**3**]. Taking into account the long follow up period of the patients, we conclude that the dacron material has very good properties: adequate filling of the removed volume, easy adaptation of final prosthesis, very good biocompatibility and long-lasting material. The encouraging results obtained with this technique for the prosthesis after enucleation of the malignant choroidal melanoma made us believe that it can also be used for enucleations of other pathologies (massive ocular trauma, sympathetic ophthalmia, and retinoblastoma).
